# Systematic Cholecystectomy During Cytoreductive Surgery Plus HIPEC: A Critical Analysis of an Empirical Tradition

**DOI:** 10.1245/s10434-024-15863-z

**Published:** 2024-07-26

**Authors:** Alida Gonzalez-Gil, Álvaro Jesús Gomez-Ruiz, Carmen Gonzalez-Pérez, Elena Gil-Gomez, Vicente Olivares-Ripoll, Jerónimo Martinez, Francisco Barceló, Pedro Antonio Cascales-Campos

**Affiliations:** 1 Departamento de Cirugía, Unidad de Cirugía Oncológica Peritoneal, Hospital Clínico Universitario Virgen de la Arrixaca, Murcia, Spain; 2 Departamento de Oncología, Hospital Clínico Universitario Virgen de la Arrixaca, Murcia, Spain; 3 Departamento de Ginecología y Obstetricia, Unidad de Ginecología Oncológica, Hospital Clínico Universitario Virgen de la Arrixaca, Murcia, Spain; 4 Servicio de Cirugía y Aparato Digestivo- Hospital Universitario Virgen de la Arrixaca, El Palmar, Murcia, Spain; 5 Departamento de Cirugía, Universidad de Murcia, Murcia, Spain

**Keywords:** Peritoneal carcinomatosis, Cholecystectomy, HIPEC, Cytoreductive surgery, Hyperthermia

## Abstract

**Background:**

Some procedures performed during cytoreductive surgery (CRS) and hyperthermic intraoperative intraperitoneal chemotherapy (HIPEC) are based on empirical data. One of these procedures is systematic cholecystectomy. This study aimed to perform a critical analysis of the need for systematic cholecystectomy during CRS+HIPEC of patients with peritoneal carcinomatosis using long-term follow-up data.

**Methods:**

Patients with peritoneal surface malignancies who were candidates for CRS+HIPEC and underwent surgery between January 2008 and December 2022 were analyzed. For patients with gallbladder involvement due to the disease or for patients whose preoperative study showed the presence of cholelithiasis, cholecystectomy was performed as part of the surgery, which was avoided for the remaining patients. All postoperative adverse events that occurred in the first 90 days were recorded, and clinical records focused on the development of biliary pathology during the follow-up period were studied.

**Results:**

The results from a consecutive series of 443 patients with peritoneal surface malignancies who underwent surgery between January 2008 and December 2022 were analyzed. The average age of the cohort was 50 years. The median follow-up period for the cohort was 41 months (range, 12–180 months), with a disease-free survival of 17 months. For 373 of the patients, CRS+HIPEC was completed without an associated cholecystectomy, and in 16 of them, the appearance of cholelithiasis was detected during the follow-up period. Only two patients in the series showed complications derived from gallstones and required a delayed cholecystectomy.

**Conclusions:**

Although cholecystectomy is a safe procedure in the context of CRS+HIPEC, it is not risk free, and its routine performance may be unnecessary.

Peritoneal carcinomatosis has been treated proactively for nearly three decades, with some cases proving to be curable.^1^ A pessimistic outlook can be countered by the fact that certain neoplasms with peritoneal spread can be effectively managed using a multidisciplinary approach that includes cytoreductive surgery (CRS) and hyperthermic intraperitoneal chemotherapy (HIPEC).^3^ As the standard treatment for pseudomyxoma peritonei and malignant peritoneal mesothelioma,^4^ CRS+HIPEC has shown benefits for patients with colorectal, gastric, and ovarian cancers.^6^ The PRODIGE 7 study indicated a survival longer than 40 months for colorectal cancer patients with cytoreduction, although HIPEC with oxaliplatin did not improve prognosis.

For ovarian cancer, combining cytoreduction with HIPEC (using cisplatin) improved survival without affecting morbidity or quality of life. The OVHIPEC2 trial currently is assessing the effectiveness of HIPEC for newly diagnosed advanced ovarian cancer. Postoperative morbidity and mortality rates are comparable with those of other major oncologic surgeries, making CRS+HIPEC beneficial for selected patients.^12^

Peritonectomy surgical procedures were standardized by Sugarbaker^13^ in the early 1990s. Performing these procedures in a regulated manner would allow complete cytoreduction of macroscopic malignant peritoneal disease. Acquiring sufficient experience with this type of surgery is associated with a long learning curve to ensure the maximum chances of survival with the fewest complications derived from the surgery.

In its original description, cholecystectomy was routinely contemplated as a step before removal of the peritoneum of the hepatic hilum and the lesser omentum during one of the peritonectomy procedures described. Many groups have maintained this recommendation, routinely performing cholecystectomy during CRS and HIPEC, even if the gallbladder is not macroscopically affected by peritoneal metastases.^14^

Although generally considered a simple and benign surgical intervention, cholecystectomy can occasionally have serious consequences, including complications that may require a liver transplantation.^15^ Morbidity varies from minor complications to complex lesions with a poor prognosis.

In Spain, the Spanish Association of Surgeons published a “do not do” document that included avoiding cholecystectomy as the first measure for individuals with asymptomatic cholelithiasis, although exceptions can include cases of hereditary spherocytosis and certain complex abdominal surgeries.^16^ In general, prophylactic cholecystectomy in non-biliary abdominal surgeries remains debated and lacks sufficient evidence for formal recommendations in most cases.

This study aimed to analyze the short- and long-term results as well as the natural history of patients treated with CRS+HIPEC for whom a routine cholecystectomy was not performed during surgery, with the intention of evaluating whether, even if the cholecystectomy is a safe procedure, it may be unnecessary.

## Patients and Methods

To analyze the natural history of patients with peritoneal surface malignancies treated by CRS+HIPEC regarding the development of complications related to the gallbladder or not in the short and long term for patients who did not have cholecystectomy performed during surgery, the study assessed a consecutive series of patients who underwent surgery between January 2008 and December 2022.

Our facility is a reference center for the treatment of complex oncologic pathologies. All the candidate patients were evaluated by a multidisciplinary committee. Every patient had adequate cardiac, renal, hepatic, and bone marrow function, and rigorous patient selection was performed using the American Society of Anesthesiologists (ASA) score. Patients with an ASA score of 4 were not selected for this procedure. The study excluded patients whose disease after laparotomy was considered unresectable. Follow-up assessment with thoracoabdominal computed tomography and tumor markers was performed every 3 months during the first 18 months, then every 6 months thereafter until the first 5 years of follow-up evaluation.

### Surgical Procedure and HIPEC Protocol

After a midline laparotomy from the xiphoid to the pubis, the tumor extension was staged using the Peritoneal Cancer Index (PCI), with values ranging from 0 to 39.^3^ All the patients followed the same operative routine, which included removal of macroscopically affected peritoneal areas and necessary visceral resections to achieve complete cytoreduction. Ostomies were limited to patients with lower-third rectal anastomoses (protective ileostomy) or preoperative fecal incontinence (definitive colostomy). Omentectomy was routinely performed. Cholecystectomy was performed only for the patients with gallbladder involvement or cholelithiasis, using a retrograde approach if necessary. Cytoreduction results were assessed using the Cytoreductive Completeness Score (CCS). The HIPEC treatment was administered only to the patients who had a CC-0 or CC-1 score, with the regimen depending on the cancer type: paclitaxel or cisplatin for gynecologic cancers, mitomycin C for colon cancer and appendix pseudomyxoma, and a combination of cisplatin and doxorubicin for other indications. The intraperitoneal treatment lasted 60 min, or 90 min for pseudomyxoma peritonei, with temperatures maintained between 42 and 43 °C using two intracavitary thermometers and one esophageal thermometer to monitor core temperature.

### Follow-up Evaluation

All postoperative adverse events that occurred in the first 90 postoperative days were collected and classified according to the criteria established by the National Cancer Institute (NCI-CTCAE version 4.0). Follow-up evaluation with thoracoabdominal computed tomography and tumor markers was performed every 3 months during the first 18 months, then every 6 months thereafter until the first 5 years of follow-up evaluation. For the patients with pseudomyxoma peritonei, follow-up evaluation was performed every 6 months until the first 3 years of follow-up evaluation, then every year thereafter.

All notes on the clinical history of the patients included in this study were retrospectively evaluated, together with the most recent available imaging tests, with the aim of defining the presence or absence of cholelithiasis during follow-up evaluation and its clinical impact (the same with its management, conservative or surgical).

### Statistical Analysis

The data were included in a prospective database established at the beginning of the surgery program at our peritoneal carcinomatosis center and analyzed using the SPSS Statistics for Windows, version 25.0 (IBM Corp., Armonk, NY, USA). We first performed a descriptive statistical analysis for each variable using the median and mean ± standard deviation for continuous variables, and frequencies and percentages for qualitative variables. Student’s *t* test, Pearson’s chi-square, and Fisher’s test were used as necessary. Associations with a *p* value lower than 0.05 were considered statistically significant. The magnitude of association between qualitative variables was calculated using relative risk. In the multivariate analysis, odds ratios were obtained, with a 95% confidence interval (CI).

## Results

The results from a consecutive series of 443 patients with peritoneal surface malignancies treated between January 2008 and December 2022 were analyzed. The mean age of the cohort was 50 years (range, 17–79 years). The main clinicopathologic variables are shown in Table 1. The most common indication for CRS+HIPEC was peritoneal dissemination caused by ovarian cancer, followed by a colorectal origin and pseudomyxoma peritonei.

**Table 1:** Main clinicopathologic variables of the patients included in the analysis

Variables	Cholecistectomy during CRS+HIPEC			
	Total (*n* = 443 *n* (%)	No (*n* = 398 *n* (%)	Yes (*n =* 45) *n* (%)	*p* Value
Age: years (range)		58 (17–79)	61 (38–78)	0.29
< 50	122 (28)	111 (28)	11 (24)	
50–70	244 (55)	220 (55)	24 (54)	
> 70	77 (17)	67 (17)	10 (22)	
Gender				0.10
Female	402 (91)	364 (91)	38 (84)	
Male	41 (9)	34 (9)	7 (16)	
ASA				0.37
1–2	292 (66)	264 (66)	28 (62)	
3	151 (34)	134 (34)	17 (38)	
Diabetes (yes)	47 (11)	44 (11)	3 (7)	0.26
Tumor origin				0.52
Ovarian	302 (68)	277 (70)	25 (56)	
Colorrectal	57 (13)	49 (12)	8 (18)	
Pseudomixoma	35 (8)	29 (7)	6 (13)	
Endometrial	13 (3)	12 (3)	1 (2)	
Sarcoma	12 (2.5)	12 (3)	1 (2)	
Gástric	8 (2)	8 (2)	0 (0)	
Others	16 (3.5)	12 (3)	4 (9)	
Previous chemotherapy (yes)	295 (66)	265 (67)	30 (67)	0.43
Previous cholecystectomy (yes)	25 (6)	25	0	–

CRS, cytoreductive surgery; HIPEC, hyperthermic intraoperative intraperitoneal chemotherapy; ASA, American Society of Anesthesiology

At the time of surgery, 25 patients had previously undergone cholecystectomy for symptomatic cholelithiasis. The main operative and postoperative variables are reflected in Table 2. The median PCI of the patients included in the analysis was 10 (range, 0–35), and complete cytoreduction of the disease was achieved for 391 (88%) of the 443 patients.

**Table 2:** Main operative and postoperative variables of the patients in the study

Surgical procedures	Cholecistectomy during CRS+HIPEC			
	Total (*n* = 443) *n* (%)	No (*n* = 398) *n* (%)	Yes (*n* = 45) *n* (%)	*p* Value
Median PCI (range)	10 (0–35)	8 (0–35)	12 (4–32)	**< 0.01**
< 10	247 (56)	231 (58)	16 (35)	
10–20	141 (32)	128 (32)	13 (30)	
> 20	55 (12)	39 (10)	16 (35)	
Intestinal resection				0.45
Colon	148 (33)	132 (33)	16 (35)	
Small bowel^a^	19 (4)	17 (4)	2 (4)	
Supramesocolic surgery (yes)	194 (43)	158 (40)	36 (80)	**< 0.01**
Right diaphragmatic	103 (23)	82 (20)	21 (47)	< 0.01
Cholecistectomy-lesser omentum	45 (10)	0	45	–
Glisson resection	45 (10)	38 (9)	7 (15)	0.15
Spleenectomy	78 (18)	60 (15)	18 (40)	< 0.01
Urinary resection	31 (7)	29 (7)	2 (4)	0.99
Abdominal wall resection	22 (5)	22 (5)	0 (0)	0.06
Ostomy				0.87
Temporal ileostomy	13 (3)	12 (3)	1 (2)	
Definitive colostomy	4 (< 1)	4 (1)	0 (0)	
Completeness cytoreductive score				**< 0**.**01**
CC-0	391 (88)	357 (90)	34 (75)	
CC-1	52 (12)	41 (10)	11 (25)	
Median surgical time: min (range)	360	300	370	**< 0**.**01**
	(190–630)	(190–610)	(290–630)	
Morbidity (yes)				0.83
1–2	81 (18)	73 (18)	8 (18 )	
3–4	71 (16)	61 (15)	10 (22)	
5 (mortality)	7 (1.6)	6 (1.5)	1 (2)	
Median hospital stay: days (range)	**7 (3–85)**	7 (3–85)	8 (5–27)	0.78
Readmision	42 (9)	41 (10)	1 (2)	0.06

CRS, cytoreductive surgery; HIPEC, hyperthermic intraoperative intraperitoneal chemotherapy; PCI, Peritoneal Cancer Index

^a^The ileum segment that is part of the ileocecal or right hemicolectomy resection is not included.

Bold values indicate *p* < 0.05

During CRS+HIPEC, cholecystectomy was part of the surgery for 45 patients (9%), due to either the presence of cholelithiasis diagnosed in the preoperative study (13 patients) or the presence of peritoneal metastases (32 patients). For 159 patients (36%), some type of complication developed, with 71 patients (16.1%) experiencing serious grade 3 or 4 complications. Seven patients (1.6%) died of grade 5 complications resulting from CRS+HIPEC. The gallbladder had not been removed from any of these patients. No patient presented with biliary complications during the immediate postoperative period of CRS+HIPEC.

Multivariate analysis of the factors associated with postoperative complications after CRS and HIPEC (Table 3), identified the following independent variables: age older than 60 years (hazard ratio [HR], 1.608; 95% CI 1.153–2.243; *p* = 0.005), need for a diaphragmatic peritonectomy (HR, 1.837; 95% CI 1.004–3.364; *p* = 0.049) or a colon resection (HR, 1.911; 95% CI 1.202–3.038; *p* = 0.006), and intraoperative transfusion (HR, 1.823; 95% CI 1.127–2.95; *p* = 0.014). The performance of a cholecystectomy during CRS with HIPEC was not significantly associated with the development of postoperative complications, even though the patients who required a cholecystectomy had a higher PCI and more frequent surgeries in the supramesocolic compartment, particularly diaphragmatic peritonectomy, and consequently a longer operative time.

**Table 3:** Multivariate analysis of factors related to the presence of postoperative complications (grade I-V) after CRS and HIPEC

	HR	95 % CI	*p* Value
Age > 60 years (yes)	**1.608**	**1.153–2.243**	**0.05**
PCI > 20	1.279	0.880–1.858	0.196
Supramesocolic compartment CRS (yes)	0.977	0.521–1.833	0.943
Diaphragmatic peritonectomy (yes)	**1.837**	**1.004–3.364**	**0.049**
Splenectomy (yes)	1.310	0.698–2.458	0.401
Colon resection (yes)	**1.911**	**1.202–3.038**	**0.006**
Blood tranasfusion (yes)	**1.823**	**1.127–2.95**	**0.014**

CRS, cytoreductive surgery; HIPEC, hyperthermic intraoperative intraperitoneal chemotherapy; HR, hazard ratio; CI, confidence interval; PCI, Peritoneal Cancer Index

Bold values indicate *p* < 0.05

**Fig. 1:**
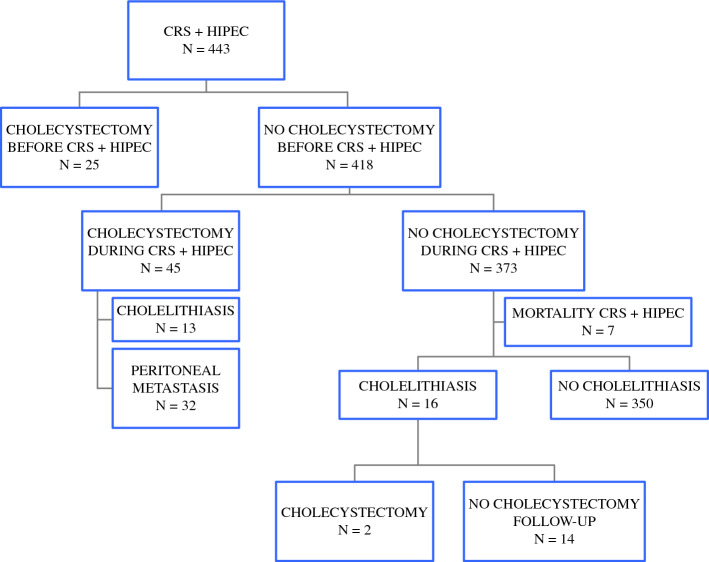
Flowchart with patient follow-up evaluation in relation to the presence or absence of long-term complications regarding the gallbladder of the patients in the study

The median follow-up period for the cohort was 41 months (range, 12–180 months), with a disease-free survival of 17 months (range, 12–180 months). A flowchart that summarizes the characteristics of the study regarding “gallbladder status” is presented in Fig. 1. For 373 patients, CRS+ HIPEC was completed without an associated cholecystectomy. From this subgroup, seven patients who had died of complications derived from surgery were excluded, resulting in 366 patients who continued with the postoperative follow-up evaluation focused on the study’s objectives.

The median time from CRS to a diagnosis of gallstones was 41 months (range, 6–78 months). The appearance of cholelithiasis was detected during follow-up evaluation in 16 patients (4%). Two (0.5%) of these patients required cholecystectomy given the presence of symptomatic cholelithiasis or complications of gallstones (1 patient with mild acute cholecystitis and 1 patient with repeated symptoms of biliary colic). For both patients, cholecystectomy was performed using an open approach, without associated complications. These two patients who underwent surgery were free of disease at the time of the indication for cholecystectomy, which was performed at 47 and 68 months, respectively.

Table 4 shows the characteristics of patients who experienced cholelithiasis during the follow-up period. For all the patients in whom cholelithiasis was detected after CRS with HIPEC, preoperative imaging studies were reviewed again, confirming the absence of visible gallstones at the time of surgery.

**Table 4:** Characteristics of the 16 patients in whom gallstones developed during the follow-up period after CRS with HIPEC

Patient	Age (years)	Gender	Tumor	PCI	Upper abdomen CRS	CCS	CRS morbidity (3–4)	DFS	OS	Time to lithiasis	Cholecystectomy (follow-up)	Status
1	61	Female	Ovary	8	No	CC-0	No	175	175	68	Yes (open), 78 months after CRS	Alive
2	64	Male	Appendix	10	Yes	CC-0	No	13	52	48	No	Deceased
3	64	Female	Appendix	23	No	CC-0	No	27	68	36	No	Deceased
4	66	Female	Ovary	3	No	CC-0	No	44	95	40	Yes (open) 47 months after CRS	Deceased
5	49	Female	Ovary	31	Yes	CC-1	No	15	88	30	No	Deceased
6	54	Female	Ovary	12	Yes	CC-1	Yes (4)	9	38	30	No	Deceased
7	45	Female	Ovary	10	Yes	CC-0	No	15	52	18	No	Deceased
8	17	Female	Mesothelioma	19	Yes	CC-0	No	50	50	48	No	Alive
9	36	Male	Colon	6	No	CC-0	No	66	66	60	No	Alive
10	78	Female	Ovary	12	No	CC-0	No	46	78	72	No	AWD
11	71	Female	Colon	1	No	CC-0	No	6	33	6	No	Deceased
12	65	Female	Ovary	3	No	CC-0	No	46	46	42	No	Alive
13	54	Female	Mesothelioma	15	Yes	CC-1	Yes (GRADO3)	47	60	36	No	AWD
14	64	Female	Colon	10	No	CC-0	Yes (GRADO4)	10	25	24	No	AWD
15	60	Female	Ovary	2	No	CC-0	Yes (GRADO3)	10	57	48	No	Deceased
16	69	Female	Ovary	4	No	CC-0	No	47	47	42	No	Alive

CRS, cytoreductive surgery; HIPEC, hyperthermic intraoperative intraperitoneal chemotherapy; PCI, Peritoneal Cancer Index; CCS, completeness cytoreductive score; DFS, disease-free survival (months); OS, overall survival (months); AWD, alive with disease

## Discussion

Our results showed that short- and long-term postoperative complications for patients with peritoneal carcinomatosis who had no cholecystectomy performed during the process are anecdotal and do not justify a systematic cholecystectomy during CRS+HIPEC, although it is a safe procedure with low morbidity rates and almost no mortality.

Since the first description of the CRS+HIPEC technique, some aspects remain highly debatable because they are based on empirical foundations without a proven scientific basis. For example, this is the case for the HIPEC method (open or closed), the performance of a digestive anastomosis before or after HIPEC treatment, the use of thoracic drains after a diaphragmatic peritonectomy without opening of the thoracic cavity, and as analyzed in this study, systematic cholecystectomy. It has not been possible to demonstrate that the administration of HIPEC by the open technique (colosseum) is better than by the closed technique. Nor does it suggest that performing a digestive anastomosis after HIPEC treatment is better in terms of locoregional control of the disease by supposedly not bathing the anastomotic surfaces than performing it before HIPEC treatment. The systematic placement of a pleural drain in patients with a diaphragmatic peritonectomy does not seem to provide any advantage over management without it.^17^ The approach of some groups regarding the systematic performance of cholecystectomy follows the same pattern of reasoning, in which the advantages of performing a systematic cholecystectomy over not performing it are not shown. This study is the first in the literature to specifically address the question whether it is necessary to perform a systematic cholecystectomy and to provide concrete data on the long-term follow-up evaluation of a non-cholecystectomized cohort during CRS+HIPEC with various oncologic indications of peritoneal carcinomatosis.

Performing a systematic cholecystectomy during CRS+HIPEC would have a series of justifications such as the possibility of a complication (e.g., postoperative cholecystitis) or long-term complications caused by the subsequent development of gallstones during the follow-up period. The parameters associated with cholecystectomy in the general population cannot be applied to this oncologic scenario. The surgery itself is responsible for the development of stasis and increased thickness of the bile, together with a decrease in motility, enhanced by prolonged fasting, fever, blood transfusion, prolonged positive fluid balance, mechanical ventilation, certain analgesics, and postoperative ileus. With the reintroduction of oral feeding after a prolonged period of fasting, the pressure on the walls of the gallbladder may increase, compromising its vascularization and triggering acute postoperative cholecystitis that may be nonlithiasic, with a greater probability of perforation. This situation is rare and can occur in any type of surgery, but is more common in abdominal surgery.^20^ In our experience of more than 15 years, we have not observed this in any of the patients in our series.

The chances of cholelithiasis development increase with age—in patients with a previous gastrectomy, in patients with extensive ileal resections, in diabetic patients, in patients with rapid weight loss, and in patients with some hematologic diseases.^21^ In other surgical settings, the same issue has arisen and remains unresolved. Although morbidity rates of almost zero associated with concomitant cholecystectomy have been described in other surgeries, some voices suggest that it could be an unnecessary procedure. For patients with gastric cancer or those undergoing bariatric surgery for morbid obesity, systematic removal of the gallbladder is not performed in daily clinical practice by all groups.^22–24^

Regarding the development of symptomatic biliary pathology in the future, studies confirm a higher incidence of cholecystitis among cancer patients, especially in the first year, although the risk remains high in the long term.^25^ The development of gallstones in our series was diagnosed in only 16 patients, and only 2 of these patients had to undergo reoperation to perform cholecystectomy for the newly appearing gallstones. These data contrast with data in a recent publication by Hanna et al.^14^ showing high complication rates related to gallstones in a heterogeneous group of patients undergoing CRS+HIPEC without cholecystectomy. Our low rates of complications related to gallstones may be explained by the fact that our surgical protocol also includes cholecystectomy for patients with preoperative gallstones.

The main limitation of this study was its retrospective nature. However, the fact that the short- and long-term morbidity rates associated with new-onset gallstones is practically nil makes it very difficult to design a clinical trial, considering that, in addition, the complications associated with cholecystectomy during CRS+HIPEC also are anecdotal.

An additional limitation given the oncologic characteristics of this type of patient was that the rates of new-onset stones could have been underestimated due to the limited survival of the patients with peritoneal carcinomatosis. After surgery, a prolonged period was necessary for the development of new gallstones, and the median disease-free survival was 17 months, after which the patients once again underwent new oncologic treatments, which displaced them to a position secondary to the problem of gallstones.

Another potential limitation was the inclusion of a cholecystectomy during CRS+HIPEC for the patients with asymptomatic cholelithiasis. We believe that the complexity of CRS, which includes surgery of the supramesocolic compartment, can create adhesions that subsequently complicate elective cholecystectomy when the gallstones become clinically apparent. This occurs in 15–20% of patients,^27^ as in our experience. On the other hand, the main strength of this study was its results for a large series of patients in a reference unit that had experience with more than 15 years of activity and the prolonged follow-up period.

In conclusion, although cholecystectomy is a safe procedure in the context of CRS+HIPEC, it is not risk free, and its systematic performance is unnecessary because in the long-term follow-up evaluation of the development of new gallstones, especially symptomatic forms, they are exceptional. The profile of patients with metastatic disease to the peritoneum who have shortened survival because of their disease also must be considered.
